# Tilapia On-Farm Welfare Assessment Protocol for Semi-intensive Production Systems

**DOI:** 10.3389/fvets.2020.606388

**Published:** 2020-11-25

**Authors:** Ana Silvia Pedrazzani, Murilo Henrique Quintiliano, Franciele Bolfe, Elaine Cristina de Oliveira Sans, Carla Forte Maiolino Molento

**Affiliations:** ^1^Animal Welfare Laboratory, Federal University of Paraná (LABEA), Curitiba, Brazil; ^2^FAI Farms, Londrina, Brazil

**Keywords:** behavior, capture, fish, health, management, slaughter

## Abstract

The aim of this study was to develop and test a tilapia on-farm welfare assessment protocol, based on Brazilian semi-intensive production systems. The study included two mains steps: the elaboration of tilapia welfare protocol and its on-field feasibility test. The protocol, including the potential indicators organized into health, environmental, nutritional, and behavioral categories, was tested on three farms. Skin, eyes, gills, jaws, fins, and vertebral spine were individually examined in 139 individual tilapias. Water physicochemical parameters and production system were considered. The overall nutritional status of individuals was assessed through body condition factor, feed conversion ratio, feed crude protein ratio, and feed ingestion behavior. During massive capture, signals of stress, level of crowding, and duration of air exposure were registered. Time required for loss of consciousness was evaluated by clinical reflexes and other behaviors during slaughter. Eye, jaw, and gill scores were different across farms (Kruskal-Wallis test, *p* = 0.011; 0.015; 0.043, respectively), showing good discrimination power. Critical welfare points were extremely low dissolved oxygen in water, fin and skin lesions, prolonged air exposure during pre-slaughter handling and non-humane slaughter techniques, as decapitation or asphyxia. The protocol presents practical viability and it is an initial step for the development of a tilapia welfare strategy, where the prioritization of critical welfare points, implementation of corrective actions and monitoring of the results is part of a permanent welfare management system.

## Introduction

In the last 20 years many studies regarding anatomic, physiologic, behavioral, and pharmacological aspects produced evidences that fish experience feelings such as pain and fear, in similar ways to other vertebrates (1, 2), as summarized in a text-book by Braithwaite (3). As evidences of fish sentience gain additional prominence (4), the concern about animal welfare by the society show parallel increases, affecting the consumer market and aquaculture regulations (5). This is so because, if fish are capable of suffering, then their welfare must be protected, within the same rationale employed for other vertebrate animals. In this context, the welfare of Nile tilapia *Oreochromis niloticus*, the most produced fish species in Brazil, may be considered a primary goal, as the number of individual animals involved is a criterium for priority in terms of animal welfare attention (6). Thus, there is an urgent need for new technologies, procedures, and strategies to detect and avoid or at least attenuate tilapia stress in all production stages and systems, so that their welfare may be improved.

In fish farming, welfare is likely to be compromised by routine management, causing stress due to the introduction of foreign objects into the water, the removal of the animal from the aquatic environment for individual restraint or underwater crowding (7). For example, the physical manipulation required for fish classification and biometrics management causes physical and psychological stress (8). In addition, water quality and associated environmental factors are areas of great attention by fish stress researchers, with water dissolved oxygen and carbon, pH and temperature and light regimes as the most critical environmental factors for maintaining fish homeostasis (9). Furthermore, there is a strong association between water quality and stocking density in fish farming systems, which is another important critical welfare point in aquaculture. When high density is associated with insufficient water renewal, the aversive effects are additive or multiplicative (1). In addition to deteriorating water quality, high density may increase aggression, lesions and disease. Under these conditions, parasitic infestations tend to thrive, generating high mortality rates (10). Additionally, restricted swimming space may also be detrimental to welfare (11). The optimal group size depends on the species behavioral characteristics and its tendency to form clusters or territorialism. Bhujel (12) suggests the optimal density of 5 animals/m^2^ for tilapia, because lower densities promote aggression between males during breeding. Barcellos et al. (13) observed that tilapia fingerlings had detrimental effects from social stress including higher cortisol levels when maintained in a stock density of 10 fish/100 L^−1^, when compared to 5 fish/100 L^−1^ and lower densities. As an additional complicating factor, the high density may influence food consumption, with dominant fish tending to eat more than others. A nutritionally balanced diet is critical to maintaining normal organic functioning and fish resistance to disease. Although periods of food deprivation may have an attenuated impact on fish balance due to ectothermia, consideration of their motivation to feed is essential in preserving welfare. Consequences of prolonged food deprivation may include aggression, erosion of the dorsal fin due to cannibalism and weight loss (14). When confined in high densities, some fish may suffer from fasting, again with potential additive adverse effects (10). Other critical welfare point is pre-slaughter and slaughter management. During massive capture, a procedure which before the recognition of fish sentience used to be named as harvest, and the transportation to the slaughterhouse, fish may suffer from multiple injuries due to overcrowding, resulting in excessive mucus production, loss of scales, damage to gill epithelium, muscle bruising and bone fractures, and extreme environmental conditions such as air and luminosity exposure as well as water quality shock. During slaughter, for a method to be considered humane it must immediately induce insensibility and be free from fear and pain (15–17). In Brazil, thermal narcosis employing ice slurry and the simplest air exposition causing asphyxia are the most common methods for tilapia slaughtering. However, these methods are not considered humane, due to the intense suffering and fear that fish experience for long periods before dying (15, 18–20).

Because of the number and diversity of welfare critical points, assessing the degree of farmed animal welfare requires the development of diagnostic techniques which are capable of considering an array of indicators. These can be classified as direct or animal-based indicators, when they are measured in the animals, or indirect or resource-based indicators, when they are measured in the environment in which the animal is inserted or relate to the management imposed on the animal (12). In addition, for the most common terrestrial farm animals, the Five Freedoms (21) are organized in robust welfare assessment protocols, such as the Welfare Quality (22) and the AWIN (23–25), which provide an organized list of specific welfare indicators to be measured and a first attempt of final integration into an overall animal welfare level. Fish-oriented scientific literature has also been building, and reported fish critical welfare points include indicators associated with feeding, water quality, sampling, capture, and slaughter (5, 7, 26). Additionally, interactions between different indicators may also present important effects on the welfare of fish.

Overall, the effectiveness of a welfare protocol depends on its validity, reliability, and feasibility. In other words, the protocol needs to be validated by expert judgment (27, 28), repeatedly achieve the same results by the same or different observers after adequate training, and be consistent in terms of required time and across different farm conditions (29). Although there are some protocols for some fish species as salmon and trout (30–33), optimal values of indicators are species-specific and no tilapia welfare protocol assessment seems to be available in the literature. Therefore, the goal of this work is to develop a tilapia on-farm welfare assessment protocol, based on Brazilian semi-intensive systems of production, bringing fish welfare assessment efforts closer to the more robust literature on the welfare assessment of terrestrial farm animals.

## Materials and Methods

The study included two mains steps: the elaboration of tilapia welfare protocol and its on-field feasibility test. For the elaboration of the protocol, initially a list of potential indicators was prepared from a literature review. Then, 12 tilapia farms in South and Southeast of Brazil were visited, for studying the measurement feasibility of each selected indicator, in terms of time and human resources required. Thus, the tilapia welfare protocol was ready for the second main step, which was its on-field testing on three different farms, chosen from our contacts with the criterium of nutritional and mortality data availability. Field results were studied in terms of each indicator potential for contributing to the overall welfare assessment and to the discriminating power across different real-life conditions relevant to the target production system.

This project was approved by the Animal Use Ethics Committee of the Agricultural Campus (No. 083/2019), of the Federal University of Paraná, Brazil.

### Elaboration of the Tilapia Welfare Assessment Protocol

The tilapia welfare assessment protocol was organized in four categories as per the literature on farm welfare assessment protocols for terrestrial animals (22): (a) health, (b) environment, (c) behavior, and (d) nutrition, considering the severity and duration of potential risks (Table 1). Health and environmental indicators were established based in salmon protocols and adapted for tilapia (Tables 2, 3) (30, 31). Tilapia environmental and nutritional needs, as per the scientific literature, were used to adjust the scores criteria. Finally, behavioral indicators were incorporated to the protocol. Scores were set for all categories, with 1 representing the desirable scenario.

**Table 1:** Health, environmental, behavioral, and nutrition indicators for the assessment of farmed tilapia welfare, based on Stien et al. (31)

	**Welfare indicator**	**Production stage**		
		**Growing/grow-out**	**Capture**	**Pre/slaughter**
Health	Eyes, jaws, operculum Skin, fins, gills Spine Ectoparasites Blood glucose Mortality Scales in water Consciousness	X X X X X X	X X X X X X	X X X X X
Environmental	Temperature, pH	X	X	X
	OD, NH_4_, NH_3_, NO_2_	X	X	
	Transparency	X		
	Stocking density	X	X	X
	Shading	X	X	X
	Predators control	X	X	
	Interspecific	X		
	Air exposure		X	X
	Light exposure		X	X
Behavioral	Gulping air at surface	X	X	
	Respiratory frequency	X	X	X
	Swimming	X	X	X
	Distribution in tank	X	X	
	Body coloration	X	X	
	Social behavior	X		
	Foraging behavior	X		
	Response to light	X	X	X
	Response to air exposure		X	X
	Loss of consciousness			X
Nutritional	Amount of feed provided	X		
	Crude protein (CP)	X		
	Feed conversion ratio	X		
	Condition factor (k)	X		
	Feeding handling	X		
	Fasting period	X	X	
	Depuration period			X

**Table 2:** Health indicators based on Stien et al. (31), scores and descriptors or reference values adapted for on-farm tilapia welfare evaluation.

**Indicators**	**Score**	**Descriptors or reference values**
Eyes	1 2 3 4	Apparently functional and healthy Hemorrhage, exophthalmos, traumatic injury; Unilateral Hemorrhage, exophthalmos, traumatic injury; Bilateral Bilateral cataract, chronic condition, impaired vision
Jaws	1 2 3	Normal aspect, healthy Light superior or inferior deformity (esthetics) Moderate superior or inferior deformity (affecting feeding)
Operculum	1 2 3 4	Normal aspect, healthy Partially covering the gills (≥75% covered) Partially covering the gills (<75%) Unilateral or bilateral absence
Skin	1 2 3 4	Normal aspect, healthy Scar tissue, scale loss, ulcers or superficial injuries <1 cm^2^ Ulcers or superficial injuries >1 cm^2^, redness, light necrosis Severe necrosis, darkening, bleeding, inflammation
Fins	1 2 3 4	Normal, healthy appearance Scarred or slightly necrotic tissue Moderate injury or necrosis (thickening/splitting) Severe necrosis, bleeding, inflammation, exposure of the rays
Gills	1 2 3 4	Normal aspect, healthy Light injury or necrosis, thickening or splitting Moderate injury or necrosis, thickening or splitting Severe necrosis, bleeding, inflammation, pallor, or darkening
Spine	1 2 3	Normal structure Lordosis or scoliosis, normal weight Lordosis or scoliosis, weight loss
Ectoparasites	1 2 3	No infestation Moderate infestation (≤5 parasites) Intense infestation (>5 parasites)
Blood glucose (mg/dL)	1 2 3 4	30–59 60–80 81–120 <30; >120
Mortality (%)	1 2 3 4	≤ 10 ≤ 25 ≤ 50 ≥75

**Table 3:** Environmental indicators based on Stien et al. (31), scores and descriptors or reference values adapted for on-farm tilapia welfare evaluation.

**Indicators**	**Score**	**Descriptors or reference values**
Temperature (°C)	1 2 3 4	25–32 20–24 33–37 ≤ 19–≥38
pH	1 2 3 4	6.0–8.5 5.5–5.9 or 8.6–8.9 8.9–10.0 ≤ 5.5 or ≥10.0
Transparency (cm)	1 2 3	25–40 41–65 <25 or >65
Oxigen saturation (%)	1 2 3 4	70–95 50–69 30–49 <30 or >95
Non-ionized ammonia (NH_3_; mg/L)	1 2 3	0.000–0.050 0.050–0.100 ≥0.100
Nitrite (NO_2_; mg/L)	1 2 3	0.00–0.50 0.50–1.00 ≥1.00
Alkalinity (mg/L of CaCO_3_)	1 2 3	30–100 20–30 or 100–200 <20 or >200
Shading (%)	1 2 3	20 a 30 31 a 40 <20 or >40
Predators	1 2 3	Absence Controlled presence Uncontrolled presence
Interspecific inhabitants	1 2 3	Absence Controlled presence Uncontrolled presence
Stocking density	1 2 3	Ideal to 10% overpopulation 10–20% overpopulation > 20% overpopulation

Ten health indicators were based on clinical examination of the eyes, jaws, operculum, skin, fins gills, and spine, the presence of ectoparasites, blood glucose, and mortality (Table 2). Environmental indicators included seven water physicochemical factors, stocking density, the presence of interspecific cohabitants, shading, and terrestrial predators (Table 3). For nutritional assessment the indicators were body condition factor, dietary crude protein level, conversion ratio, and feeding behavior (Tables 4, 5). Condition factor (K) was defined as K = (WL^−3^)100 to estimate tilapia nutritional status, where W is the weight (g) and L is the length (cm) (12, 27). The K factor was classified according to the value obtained, with score 1 for K between 1.6 and 1.9; score 2 for 1.1–1.5 or 2.0–2.3; and score 3 for ≤ 1.0 or ≥2.4. Stocking density was also relevant for nutritional evaluation, which was classified as adequate or inadequate according to life stage recommendations (34). For all nutritional indicators, score 1 was the ideal scenario, 2, 3, and 4 being off in 10, 20, and more than 20% of optimal values, respectively. Feeding behavior was classified as appropriate if fish consumed the feed within 3–5 min. The swimming behavior and the level of fish body air exposure during capture, and the time for the loss of consciousness after stunning or slaughter procedures were also included in the protocol (Table 5). The indicators for the evaluation of tilapia consciousness included the clinical reflexes: opercular rate (OR), vestibulo-ocular reflex (VOR), equilibrium (EQ) and the tail-grab-reflex.

**Table 4:** Different densities considered ideal according to the association between raising system and tilapia feed conversion ratio (FCR) and diet crude protein rate (CP), adapted from RSPCA (33).

**Raising system**	**Weight (g)**	**Age (days)**	**Stock density (fish/m**^****2****^**)**		**FCR**	**CP (%)**
			**No aeration or renew**	**Aeration or renew**		
Excavated pond	1–30	40–80	20–30	40–50	0.8–1.0	36-40
	30–200	80–120	4–5	6–10	1.2–1.3	28–32
	200–1,000	>120	0.8–1.2	2–3	1.4–1.6	28–32
Cage	1–30	40–90	1,200–1,500		0.8–1.0	40
	30–200	90–120	450–500		1.2–1.4	32
	200–1,000	>120	100–150		1.6–2.0	32

**Table 5:** Scores used to classify feeding, capture, and slaughter indicators and respected characteristics for on-farm tilapia welfare assessment, based in Noble et al. (26).

**Management**	**Score**	**Criteria**
Feeding	1 2 3 4	Apprehension of all food in 180–300 s Apprehension of all food in 120–180 s Apprehension of all food in ≤ 120 s No apprehension of all food or ≥360 s
Capture	1 2 3 4	Normal swimming, no or low dorsal fins or body parts on surface Excited swimming behavior, >20 dorsal fins or low body parts on surface Swimming in different directions or decreasing activity, fish stuck against net Many fish floating on side, explosion of body to air, exhaustion
Slaughter	1 2 3 4	Instantaneous loss of VER, BO, EQ, and TGR Total loss of VER and BO in ≤ 10 s, instantaneous loss of EQ and TGR Total loss of VER and BO in ≤ 100 s, instantaneous loss of EQ and TGR Total loss of VER and BO in ≤ 1,000 s, progressive loss of EQ and TGR

### On-Field Feasibility Test of the Tilapia Welfare Assessment Protocol

The welfare was assessed using potential indicators during summer (December, 2019–March, 2020) at two different grow-out excavated pond farms (A and B) located in Joinville, Santa Catarina, and one grow-out tilapia cage facility (farm C) in Guaíra, São Paulo, respectively, in Southern and Southeastern Brazil. Farm A was composed by six rectangular excavated ponds built on flat ground, and the assessment of water quality and massive capture was performed in two of them, with areas of 3.385 and 5.050 m^2^. The circulation system was maintained by a diversion canal and the water flow was controlled through a water intake and drained by a drainage canal. On farm B there was one 2.115 m^2^ excavated pond, that was supplied from the water-table by seepage into the pond. On farm C there were six excavated ponds as well as fish cages placed inside them or directly in a river channel. For farm C, the study was performed in two different excavated ponds of an area of ~10.000 m^2^, each containing 28 and 42 floating steel fish cages of 4.8 m^3^ (2.0 ×2.0 ×1.2 m) with a 20 mm steel galvanized mesh covered with PVC and four floaters: The water was renewed both indirectly by gravity and pumping through a diversion canal from a reservoir. There was were an individual inlet and an individual an outlet for each pond.

Indicators were measured always by the same researcher, during each farm routine schedule and with minimum interference to the daily management and procedures. The samples sizes were defined according to the farm dynamics, assessing the maximum number of individuals without disturbing farm routine. On farms A and B, it was possible to access a larger sample, derived from massive capture. On the other hand, due to the complexity of tests performed on farm C, where slaughter was performed, the number of individuals was reduced, avoiding disturbances to the slaughter processing line. In total, 139 tilapias were physically scored: 72, 40, and 27 animals on farms A, B, and C, respectively. Fish were removed from water, placed in a desk covered with soft material, identified using numbered waterproof cards, which were placed beside the animal for bilateral photographic registration. Sequentially, the tilapias were weighed, measured, and physically assessed according to the scores set in the protocol. On farms A and C, fish were in the end of final grow-out stage phase and thus destinated to slaughter after individual scoring. On farm B, as tilapia were in the intermediate grow-out phase, they were returned to the pond of origin.

All the environmental indicators were assessed minutes before removing fish from water for massive capture (farms A and B) or for biometrics (farm B). The physicochemical indicators were measured directly in the water by insertion of an equipment for multi-parameter measurement (AK 87, Akso, Brazil). The depth of 30 cm was standardized for measuring water temperature, pH and dissolved oxygen (DO). Water samples were collected for conducting the colorimetric test of total ammonia (NH_4_), nitrite (NO_2_) and alkalinity (Acquacombo TD 1555, Alfakit, Brazil), tested immediately after collection. Non-ionized ammonia (NH_3_) was estimated using a specific formula considering water temperature and pH (35).

Feeding behavior was measured considering the time taken for the food to be fully consumed by the animals. Production indices were collected via interview with farm staff, mostly regarding mortality rate, stocking density and daily amount of feed provided, to calculate feed conversion rates.

The welfare score of massive capture was assessed once on farms A and C, according to the capture method adopted in each place, which directly influenced the length of the light and air exposure periods, as well as the level of crowding (Table 4). On farm B, the animals studied were those captured for farm routine fish biometric procedure, using a fishing net. According to the husbandry normally adopted on farm C, after the capture fish were weighed in groups of 20 animals and individual weight was estimated by the group mean. For this reason, it was not possible to measure the fish length, so the K factor was not calculated for the animals studied on farm C.

After massive capture, the slaughter score was assessed in a slaughterhouse attached to farm C. Health exam and blood glucose analysis were made before fish slaughter. Blood was punctured from caudal vein using a 25 ×7 mm needle coupled to a 3 mL syringe. Blood was then transferred to a glucometer strip for instantaneous glucose measurement (AccuCheck Active, Roche, Brazil). In sequence, the slaughter technique was assessed, based on the registration of the time needed for the loss of consciousness (36). The OR is the simplest way to estimate the respiration rate, by counting the opercular movement, which is inversely proportional to the level of consciousness. The VOR is measured by the visual evoked response (VER) or “eye roll,” that is the eye movement when fish body is rolled from side to side through the vertical axis (37). The EQ was evaluated by fish position and its swimming capacity when placed into the water. TGR is the grabbing of the animal's tail to verify whether the fish attempts to escape (26), being an effective way to evaluate the capability of fish to interact with the external environment (15).

### Statistical Analysis

Descriptive analysis was used to estimate the welfare scores obtained during the field evaluations. The normality of data was tested by Shapiro-Wilk test (*p* < 0.05), using Statistica Statsoft 10.0. *T*-test was applied to compare farms A and B in relation to factor K results, because it was the only variable with normal distribution (*p* = 0.054). The non-parametric results obtained for health indicators on farms A, B, and C were compared by Kruskal-Wallis, aiming to detect some uniformity of results in relation to the critical welfare points observed in different facilities. Correlation analyses (*p* < 0.05) were conducted aiming at an enhanced comprehension of the relationship between environment conditions and health results.

## Results

### Health Indicators

**Figure 1:**
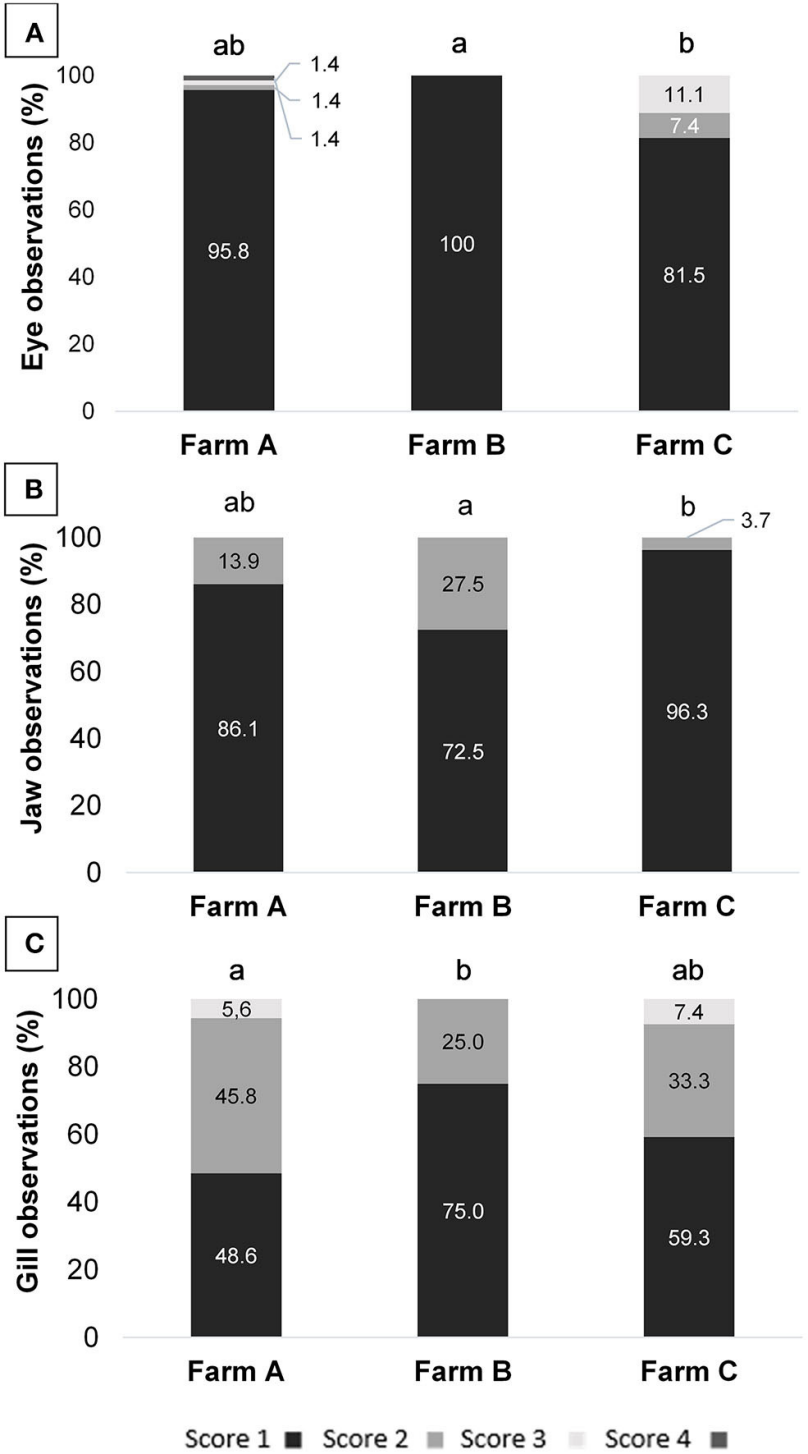
**(A)** Eye, **(B)** jaw, and **(C)** gill scores assessment of tilapia from three different fish farms; different letters indicate significance obtained by Kruskal-Wallis test (*p* = 0.011; 0.015; and 0.043, respectively).

Health indicator results are summarized in Table 6. When comparing scores from different farms, differences were observed for eyes, jaws and gills (Figure 1). On Farm A, few animals presented damage in different degrees of severity in the eyes, being opacity, unilateral, and bilateral hemorrhage. From the total of 72 animals examined in that farm, 10 tilapia (13.8%) presented damaged jaws and two had unilateral partial loss of operculum. Spine seemed to be healthy in most animals, with just one case of scoliosis. Also, when evaluating ectoparasites, just one parasitic copepod commonly called anchor worm *(Lernaea* sp.), attached to the skin was detected. Light gill lesions without color alteration were observed in 33 fish (45.8%); five animals had moderate lamella fusion and excessive mucus production. From that, 36 animals (50.0%) showed splitting of caudal fin, and seven of these had additional necrotic dorsal fins. The main alteration observed in skin was subcutaneous hemorrhage, usually located in fish face between snout and operculum. On farm B eye damages, vertebral deformations or ectoparasites were not registered. However, from the 40 tilapia, 11 animals (27.5%) showed jaw lesions and 14 (35.0%) presented red spots on other areas of face skin. Gill splitting and excessive mucus production were observed in 10 fish (25.0%). Caudal fins of 19 animals (47.5%) were splitted and one tilapia presented dorsal fins light necrosis (2.5%). On Farm C, one tilapia (3.7%) from the total of 27, presented lesions on the jaw and operculum. When examining the eyes, two animals (7.4%) had unilateral hemorrhagic lesion and three animals presented bilateral exophthalmos. Gills of nine animals (33.3%) showed partial damage (lamella fusion) and two animals (7.4%) exhibited severe damage, including two positives for parasites with macroscopic signals suggestive of the monogenoid *Dactylogyrus* sp. Future work may include microscopic diagnosis of eventual parasite species. As for the fins, 21 animals (77.8%) presented light erosion of one or more fins, and one of them presented severe necrosis (3.7%). Coherent with clinical findings for the fins, when examining body skin, different alterations were observed, as lack of scales (*n* = 7; 25.9%), ulcerations (*n* = 2; 7.4%), necrosis (*n* = 2; 7.4%), and body skin darkening (*n* = 1; 3.7%). No skeletal deformities were detected. Blood glucose level, measured on farm C immediately before slaughter, was 86.44 ± 13.75 mg/dL, distributed in scores 1 (5.6%), 2 (27.8%), and 3 (66.7%). According to farm staff data, the mortality along the production cycle on farms A and C was 5 and 10%, respectively, and both were classified as 1; on farm B reported mortality was 15%, thus equivalent to score 2.

**Table 6:** Health scores (%) and *p*-values in three different tilapia grow-out farms, data collected from January to March 2020 in South and Southeast Brazil; comparison amongst farms with Kruskal-Wallis test for all indicators.

**Health indicator**	**Farm A** ** (*****n*** **= 72)**				**Farm B** ** (*****n*** **= 40)**				**Farm C** ** (*****n*** **= 27)**				
	***1***	***2***	***3***	***4***	***1***	***2***	***3***	***4***	***1***	***2***	***3***	***4***	***p***
Eyes	95.8	1.4	1.4	1.4	100	0.0	0.0	0.0	81.5	7.4	11.1	0.0	0.011
Jaws	86.1	13.9	0.0	–	72.5	27.5	0.0	–	96.3	3.7	0.0	–	0.015
Operculum	97.2	2.8	0.0	0.0	100	0.0	0.0		96.3	3.7	0.0	0.0	0.475
Gills	48.6	45.8	5.6	0.0	75.0	25.0	0.0	0.0	59.3	33.3	7.4	0.0	0.040
Skin	52.8	36.1	11.1	0.0	65.0	35.0	0.0	0.0	55.6	25.9	14.8	3.7	0.311
Fins	40.3	50.0	9.7	0.0	50.0	47.5	2.5	–	18.5	77.8	3.70	–	0.056
Spine	98.6	1.4	0.0	–	100	0.0	0.0	–	100	0.0	0.0	–	0.543
Ectoparasite (*Lernaea* sp.)	98.6	1.4	0.0	–	0.0	0.0	0.0	–	100	0.0	0.0	–	0.543

### Environmental Indicators

The excavated pond areas were 3.385 and 5.050 m^2^ on farms A and B, respectively. On farm C, an excavated pond accommodated 28 tilapia cages measuring 4.8 m^3^ (2.0 ×2.0 ×1.20 m), totaling 134.4 m^3^ of area available to the animals. Even though stocking density was not considered elevated, farms A and B presented critical levels of DO, of 1.6 and 1.7 mg/L respectively (Table 7). On farm A the pH was 6.5, the limit of water acidification considered adequate for tilapia, probably related to low alkalinity and high transparency of water. In all scenarios, few concerns were observed about the pond's external environment. In this way, unsatisfactory scores were observed in relation to the absence of shading. Also, it was detected uncontrolled presence of terrestrial predators, mainly birds in farm A and B. In farm C fish were protected by a 25 mm galvanized steel screen cover, avoiding predation. Despite that, many birds were present above it and in land near cages. The maintenance of tilapia as a unique species was adopted in all evaluated systems, so aquatic predators or competitors were not detected.

**Table 7:** Environmental indicators values and scores of three different tilapia grow-out farms, data collected from January to March 2020 in South and Southeast Brazil.

**Environmental indicator**	**Farm A**		**Farm B**		**Farm C**	
	**Value**	**Score**	**Value**	**Score**	**Value**	**Score**
Temperature (°C)	28.0	1	26.5	1	29.8	1
pH	6.5	1	7.0	1	7.5	1
Transparency (cm)	31.0	2	28.0	1	22.0	1
DO (%)	19.0	4	21.0	4	50.8	2
NH_4_ (mg/L)	0.060	–	1.821	–	0.815	–
NH_3_ (mg/L)	0.001	1	0.011	1	0.019	1
NO_2_ (mg/L)	0.000	1	0.000	1	0.000	1
Alkalinity (mg/L)	20.0	2	30.0	1	40.0	1
Shading (%)	0.0	3	0.0	3	0.0	3
Predators	UP^a^	3	UP^a^	3	CP^b^	2
Inhabitants	NI^c^	1	NI^c^	1	NI^c^	1
Density (^d^fish/m^2^; ^e^fish/m^3^)	1.3^d^	1	3.6^d^	1	70.0^e^	1

a Uncontrolled presence;

b Controlled presence;

c Non interspecific inhabitants.

d fish/m2;

e *fish/m3*.

### Nutritional Indicators

In all scenarios, fish were self-fed twice daily with commercial extruded pellets. Even for fish maintained in the pond for an extended period on farm A, according to stocking density informed by staff and the amount of feed provided, the FCR calculated was considered adequate (Table 8). In farm B, the crude protein ratio was 6% higher than the suggested for weight and age criteria, being classified as welfare score 2. K factor means for farms A and B were 1.52 ± 0.50 and 2.45 ± 0.50 (*p* = 0.000), scored as 2 and 3, respectively.

**Table 8:** Nutritional indicators and related information for three different tilapia grow-out farms data collected from January to March 2020 in South and Southeast regions of Brazil.

**Technical information and nutritional indicators**	**Excavated pond**		**Cage**
	**Farm A**	**Farm B**	**Farm C**
Fish weight (g) (mean ± SD)	737.9 ± 132.6	274.2 ± 39.26	1080.5 ± 229.8
Fish age (days)	418	118	400
Density (^a^fish/m^2^; ^b^fish/m^3^)	1.30^a^	3.61^a^	70.00^b^
Crude protein ratio (CP) (%); score	32/1	38/2	32/1
Feed conversion ratio (FCR); score	1.45/1	1.54/1	2.00/1
K factor (mean ±SD); score	1.52 ± 0.50/2	2.45 ± 0.50/3	–
Feeding (min); score	5 min/1	5 min/1	1 min/3

a fish/m2;

b fish/m3.

Farms A and B adopted similar protocols for delivering the feed, where handling was made manually from just one pond margin side. The superficial swimming efforts of fish in the direction of the feed pellets were noticeable. Farm A had a larger swimming area, being difficult for all the fish to achieve the pellets during feeding time. In both situations, the consumption occurred within 5 min. Feed management on farm C cages was carried quickly by canoe, taking around 20 min to cover all the 28 cages. The feed intake was quite fast, ~1 min for total feed consumption. This may be an indicative of underestimation of the amount of feed to offer, especially for days of intense heat, when the metabolism of the fish is accelerated. However, an excess of feed was observed in cages near the margins, probably due to low consumption associated with the stress of massive capture, as fish caught and considered underweight, called rejects, were reallocated into these same cages.

### Behavioral Indicators

**Figure 2:**
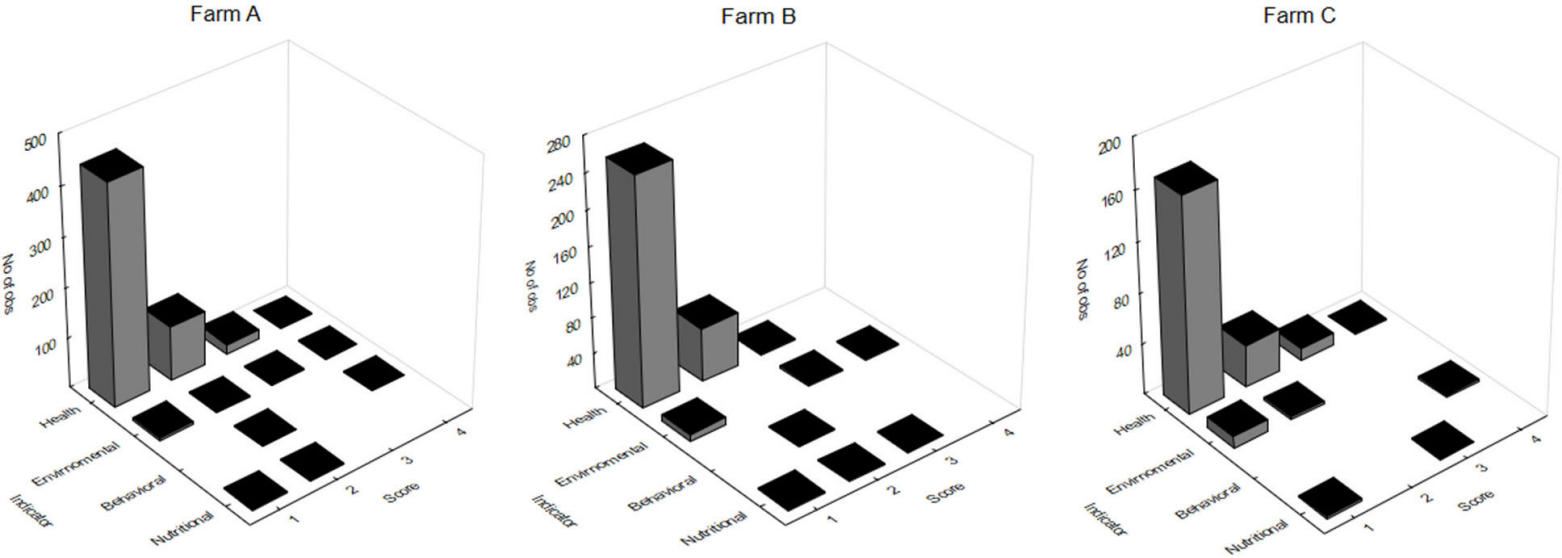
Tilapia welfare scores obtained for health, environmental, nutritional, and behavioral indicators in three different farms **(A-C)**; no significance was found when comparing farms (Kruskal-Wallis; *p* = 0.435).

The massive capture occurred between 7 h 00 and 7 h 30 a.m. in all scenarios, which likely provides less stress than would be caused by the stronger light and heat of the most advanced hours of the day. On farm A, fish were caught by five men pulling a fishing net from inside the pond. The procedure lasted more than 3 h. After collection, animals remained overcrowded and stuck in the fishing net, being exposed to sun light for around 30 min, until being gradually removed from water manually. Most fins and body parts could be observed over the water column and exhaustion was evident through the intensive swimming as tentative to escape. Fish were then placed in dry plastic boxes (35 animals/box) and placed in 1,000 L transport boxes located over a fish transport truck, containing water and ice. On farm C the procedure was faster, as the massive capture, including the cage displacement to the handling deck and lifting, the fish capture by fishing net (~10 fish/catch) and weighing lasted 20 min. However, some critical welfare points in relation to this management were observed. Fish that were not sold to customers immediately remained in same cage until slaughter time by the afternoon. In the meantime, the water column was extremely reduced due to the tank lifting. Fish presented agitated swimming and some parts of body were exposed to air and luminosity. In farm B the capture using fishing net was performed four times, aiming to collect enough animals to conduct biometrics. Fish were placed in 10 L bucket (1 fish L^−1^) containing pond water for ~15 min. Even if its duration was faster than the massive capture observed in other scenarios, animals from farm B showed attempts to escape and acceleration of opercular beating. Considering the period of capture, handling, and its consequent air exposure and crowding, farms A and B were classified as score 2 and Farm C was classified as score 4. As for pre-slaughter and slaughter, on farm A fish were placed in crowded transport boxes containing water and ice until transport to the slaughterhouse. No control of temperature, DO or stunning effectiveness was observed. In the first minutes after allocation in the transport container, animals presented agitated swimming and frequent escape behavior, and at the end of the capture procedure the first animals to be submitted to the ice slurry were apparently dead. On farm C, 10% of animals were sold directly to the local market. In this case, the tilapias were placed in raffia bags without any procedure for slaughtering or stunning them, and consequently probably died from asphyxiation on the way to the reseller. The animals considered too small were thrown alive to be consumed by birds, constantly present around the tanks likely conditioned to that practice. Remaining fish, on farm C, were placed in 500 L tanks containing water and arranged in a truck; after arrival at the slaughterhouse complex, the animals were transferred to another tank. This slaughterhouse tank had a recirculation pump; however, there was no filtering system or temperature control device. Animals that were slaughtered lastly showed signs of physical exhaustion, apathy and remained practically outside of the water because the tank was drained before the slaughter procedure was complete. On the processing table, tilapias were decapitated with a knife and fileted. It was noticed that after decapitation, some organs as the heart and pectora fins stayed connected to the fish head. This allowed for the presence of movements which characterize consciousness in most severed heads. From 10 animals that were evaluated, severed heads presented frequent OR, six showed attempts to swim when reconditioned into water and three showed mouth regular opening movements. The average time for loss of OR and VER were 257.36 ± 121.42 and 301.87 ± 120.16 s, respectively. However, no fish showed a reaction to the pain stimulus applied in the lips after decapitation. Due to the prolonged suffering that animals were exposed to in both scenarios, slaughter welfare score was classified as 4. In total, 26 welfare scores were measured on farms A and C, and 25 evaluated on farm B, including health, environment, nutrition and behavior indicators. For the comparison between farms analysis, all indicators scores were included. Despite the high frequency of score 4 on farm A, when considering the total added welfare scores, no significance between farms was found (Figure 2; *p* = 0.435). Results of analyses showed a weak correlation between gill score and weight (Table 9). The K factor was moderately correlated to DO and pH. No significance or very weak correlations were observed between health indicators (gills, fins, and skin) scores and environmental indicators.

**Table 9:** Correlation between different health, nutritional and environment indicators of tilapia welfare, showing *p*-value, coefficient of determination (r^2^), tendency and correlation strength.

**Variable**	**Gills**	**Fins**	**Skin**	**K factor**
Weight	0.01; 0.07; (+) VW	0.09; 0.03; (+) NS	0.55; 0.00; (+) NS	0.00; 0.34; (–) WK
DO	0.47; 0.00; (–) NS	0.20; 0.01; (+) NS	0.06; 0.03; (+) NS	0.00; 0.41; (+) MD
Temperature	0.99; 0.00; (+) NS	0.42; 0.00; (+) NS	0.04; 0.03; (–) VW	0.00; 0.24; (+) WK
pH	0.13; 0.02; (–) NS	0.14; 0.02; (–) NS	0.16; 0.01; (–) NS	0.00; 0.51; (+) MD
NH_3_	0.17; 0.01 (–) NS	0.89; 0.00, (+) NS	0.23; 0.01; (–) NS	0.17; 0.02; (+) NS

*Correlation strenght based on r^2^ range: (VW) Very Weak: 0.00–0.19; (WK) Weak: 0.20–0.39; (MD) Moderate: 0.40–0.69; (ST) Strong: 0.70–0.89; Very Strong (VS): 0.89–100; (NS); Not significant (p > 0.05)*.

## Discussion

To improve animal welfare in fish farming standardized protocols to assess welfare are needed (38). The main objective of this work was to develop a tilapia welfare assessment protocol and test its discrimination power to prioritize critical welfare points as well as to show differences amongst real life situations. The robustness of the protocol was possibly increased due to the observed variability across farms, as an extensive list of indicators was included, making the protocol more suitable for generalization to an array of realities. Even then, this is a preliminary protocol to which more indicators will likely be added in the future, as shown in the case of species in which animal welfare assessment has been studied for longer [see for example Souza et al. (39)].

The quality of the data reported by local staff, mainly regarding stocking density and mortality rate, is a relevant factor to consider, as the lack of accurate information can preclude the calculation of the adequate animal sample size for individual evaluation and, consequently, a precise welfare assessment. For example, the mortality considered here refers to the total percentage recorded over the grow-out cycle, i.e., historical mortality. Rates of mortality vary considerably depending on the production stage and farm management adequacy, with 20–71% mortality reported for tilapia, and is an important tool for the identification of critical welfare points identification (40). However, in addition to the lack of this indicator usually observed on small farms, a low mortality rate does not guarantee the absence of disease (26) or a high degree of welfare (5).

Results of health indicators showed important variability in gill, fin, and skin conditions. Gills are vital organs, and as they are exposed to the external environment, their changes are generally visible and can indicate various diseases such as parasitosis, bacteriosis, and viral infections (30). Gill aspect is also considered an important indicator of water quality, and its alterations may reflect signals of inadequate pH or intoxication by high levels of ammonia, nitrite, or chlorine (41). In addition, when subjected to prolonged air exposure, gill lamellae collapse and adjacent filaments adhere, reducing the gas exchange area and causing hypoxia (42). This gill collapse or division was a common alteration observed during health evaluation in farms A and C, after massive handling, indicating this excessive air exposure during the procedure.

Similarly, fin alterations may be a signal of various diseases, inappropriate handling or cannibalism. The relationship between severity, frequency and type of fin damage and welfare is not well-understood (5, 26). However, fins are composed of hemispheric tubes containing blood vessels and nerve bundles of nociceptors; therefore, a fin lesion may be painful (43). Also, fin damage may have a detrimental effect upon growth and survival, and may potentially reduce swimming ability, affecting fish welfare (19). In this way, it is important to determine if the lesion is acute, being recently induced by the management itself, or chronic. Fin chronic erosion can occur as a primary or secondary consequence of bacterial or fungal disease (44). In farms A and B, it was possible to observe high prevalence of caudal fin splitting, operculum damage, and redness in the skin of the tilapia head, all characteristic lesions of capture using knotted nets (45–47). The tendency toward a significant result when comparing fin conditions amongst the three farms pointed to this influence of capture management, explaining the higher splitting occurrence in farms A and B. Differently, the injuries with higher prevalence and severity observed in farm C, are characteristic of bacterial necrosis. Erosions in fins can also be caused by turbine pumps and crowding during capture, feeding handling, water temperature and oxygen supersaturation, exposure to light and consequent sunburn (19, 26). The preventive measures include the use of knotless netting or vacuum pumping for fish transference, and the use of demand feeding technology, avoiding cannibalism (19, 26, 44). Specific and more detailed investigations of types of lesion and on which fins they usually occur are recommended for tilapia welfare evaluation, being an important tool for farmed fish welfare improvement.

The significant difference observed for eye scores between farms may relate to the sanitary condition of each property. As eye alterations are indicative of several pathologies, this indicator presented good discriminatory power, exposing the problem observed in farm C. Ocular disorders in fish are common and may occur as primary or secondary manifestations of a systemic disease (48). Exophthalmos is a clinical sign of important bacterial pathologies that affect tilapiculture, such as *Aeromonas hydrophila, Streptococcus agalactiae, Flavobacterium columnare* (49), and orthomyxo-like virus, the agent of tilapia lake virus disease (TiLV) (49–51). These viral and bacterial co-infections are common (50). Many of the skin damages observed during our assessment, such as hemorrhages, ulcers and body darkness, were also compatible with bacterial infections, mechanical injuries or stress caused by handling. The fish epidermis is a multifunctional organ with highly relevant physiological roles, including a cutaneous stress response that protect the organism against unfavorable conditions (52). If this barrier is physically lesioned, the organism is more susceptible to infections. Additionally, the skin is loaded with nociceptors, and thus every damage potentially painful, considering lesion frequency and severity. Overall, epidermal damage is easy to evaluate and an important welfare indicator as it indicates serious welfare concerns (44). It also revealed important prevalence, with scores 2 and 3 in all real scenarios studied.

Despite the significance amongst farms, jaw evaluation evidenced an acute welfare problem, as the lesions seemed to be resultant from the net crowding during massive capture (45). Another indicator that showed low prevalence was the presence of ectoparasites. However, parasitic diseases are among the most frequent problems in aquaculture and are frequently associated with inadequate water conditions and high densities (42). Tricodinids and monogenoidea are among the most important ectoparasites in tilapia (53–55), with high parasite specificity to the gills and skin during the warmest months, causing discomfort and death by asphyxia (53, 56). However, their definitive diagnosis is only possible through the microscopic analysis. As the intention is to develop an on-field protocol to be executed by farmers, alternatives to laboratory analyses are preferred (22, 31, 57). Bui et al. (58) proposed behavioral observation as an indicator of Atlantic salmon sea lice infestation, as the increase in flashing or jumping behavior are potential signals of higher parasite prevalence. In addition, the behavior may be assessed by farmers through standardized visual surveillance or through more advanced methods such as assessment of shoaling behavior from video recordings (58). As ectoparasites represent a critical fish welfare issue, affecting locomotion, competition skills, and foraging behavior (59), the development of specific behavioral measures for the assessment of responses to parasites in tilapia is relevant to the management of their welfare.

Blood glucose is an invasive measurement when dealing with tilapia, considering the air exposure, hemorrhages and skin lesions that may occur and carry on relevant risks for the future welfare of the animals, such as for instance the development of infections. In this work, fish were sampled immediately before slaughter due to these risks. In addition, the procedure is time consuming for handlers, requiring specific skills that may not be common for farm staff. Furthermore, a set of rapid, inexpensive and non-invasive screening methods is preferable as on-field welfare indicators (38). It is also fundamental to consider that glucose levels in this setting represent the intensity of the stress related to massive capture, transportation, and other pre-slaughter interventions, being less related to the stress levels during life. This indicator is more meaningful if compared with pre-stress levels rather than any standard levels, as plasma glucose is also dependent on feeding status, diet type, and other factors (26). However, overall glucose levels may convey important information in terms of severe stress. According Martínez-Porchas et al. (60), glucose cannot be eliminated from a list of stress indicators, preferably when evaluating a chronic exposure to stressful conditions, but must be complemented with other stress measurements as hormones or blood-cell counts, in order to have a more complete profile about the stress status of any fish.

High rusticity is associated with tilapia, involving ample tolerance to a wide range of temperature and pH. Thus, predictably, most results for environmental indicators were within acceptable levels for the species. However, DO on farms A and B was critically low. Despite the species strong ability to survive a few hours even under anoxia, DO is one of the limiting environmental factors for tilapia (61). The concentration of DO in water is influenced by water temperature, salinity and atmospheric pressure (62), and the solubility of oxygen decreases as temperature increases (63). Ross and Ross (64) reported that tilapia handling increased DO consumption from 150 to 300% and that the high temperature also increases the oxygen consumption. All these factors may have contributed to the extremely DO low rates observed in farms A and B. Therefore, specific stress factors must be avoided in warm periods. Also, in the three scenarios, it was difficult to establish water renewal rates, as there was practically no inflow, due to the abnormally dry summer that occurred in the South and Southeast regions of Brazil. The chronic exposure to low DO causes fish immunosuppression and performance reduction (51), affecting welfare direct and indirectly. When DO reaches 45 to 50% of saturation (3.0–3.5 mg/L, at 28–30°C), tilapia reduces it metabolic activity as a regulation mechanism, reducing respiration and growth, and in saturations between 10 and 20% (0.7–1.6 mg/L at 26–35°C) generate great discomfort and eventual mortality (34, 65). The chronic stress associated with the metabolic reduction caused by the low oxygen availability may affect fish growth (66), which may explain the negative correlation we observed between DO and K factor. Normally, in Brazil the tilapia production cycle occurs in 6–9 months, with a target body weight of around 800 g (34, 67); however, in farm A, the animals were older than 12 months. The very low DO and chronic stress related to this indicator may have interfered in farmed tilapia growing ratio. Slow growth was also observed on farm C, probably due to malnutrition caused by inadequate food distribution, aligned with the observation of fast feed consumption in most cages. On farm B, slow growth rate was not observed, despite a low oxygen level, a result likely related to the young age of the animals and, consequently, no possibility for the evaluation of growth rate within a longer period of time. In addition, on farm B fish were apparently well-nourished, based on fish weight and K factor, and the feed was provided in adequate quantities. The K factor was an important indicator of nutritional status with discriminating power between different growth stages of farms, as it is inversely proportional to fish length, explaining the significance between farms A and B. Standard K factor for tilapia was not found in the literature; however, our results seem coherent with previously reported values. Ighwela et al. (68) reported K factor varying between 1.64 and 1.79 for tilapia fingerlings of 14.52 ± 6.39 g fed on different maltose levels; Anani and Nunoo (69) founded a K factor of 2.01 for fish weighing 140.3 ± 23 g when consuming a specific formulated diet. These results also show variation in K factor according to fish development stage, a characteristic that requires attention.

The economic demand for a short production cycle and fast growth rate was likely related to the high protein levels in the fish diet. However, Mengistu et al. (40) showed that tilapia FCR decreased with increasing CP, DO, and pH. Thus, this management may negatively affect fish FCR and their weight gain. Excessive protein levels result in additional energy expenditures, as excess amino acids require metabolization (70). The integrated production system adopted in the State of Santa Catarina, in which the slaughterhouse supplies the fingerlings and the feed to producers, may influence feeding decisions, as producers are exempt from feeding costs, but committed to delivering fish for slaughter in a short period of time. Decisions regarding feed distribution management are also extremely important (1). When feed is offered exclusively from one pond margin side, a privilege based on behavioral dominance is favored, with the larger animals becoming better fed and the smaller animals prevented from accessing adequate amounts of feed (71). In cages, fish that are located in the superficial water column tend to be benefited (71). This is one of the reasons for unevenness in fish weight and the occurrence of many rejected animals on farm C. When animals cannot satisfy their motivation for feeding, their welfare is compromised. As a source of additional secondary welfare problems, underfeeding tends to increase agonistic behavior due to the intense competition for resource, which may result in injuries (19). The use of demand-feeders spread on the pond margins reaching different depths in the case of cages, may be an alternative for underfeeding (51). Nevertheless, to ensure adequate nutrition, it is necessary to study the distribution of tilapias in the water column and the fish dominance behavior during feeding.

During the catabolism of ingested proteins, fish produce nitrogenous waste which is excreted through urine. The main end product of such catabolic activity is ammonia, which is toxic for fish. Ammonia is also derived from decomposition of organic material such as feed leftovers, feces, and organic fertilizer (72). However, its toxicity depends on other water physicochemical parameters, mainly pH and temperature (73). As pH increases above 7.0, a greater percentage of total ammonia is converted from the ionic form (NH_4_) to the toxic un-ionized gaseous form (NH_3_) (74). In addition, ammonia is more toxic at higher temperatures (75). Despite the accumulation of feed in some locations on farm C and the low water renewal, high values of NH_3_ and NO_2_ were not observed. As on farm A and B pH was acid and neutral, respectively, the ammonia value registered did not represent risk of toxicity.

Low alkalinity observed on farm A is associated with water acidification, due to the lack of buffering capacity of the system. When bicarbonates and carbonates are maintained in satisfactory levels, the pH tends to be stable, avoiding fish acidic or alkaline stress (51). The total alkalinity of water tends to be higher with the presence of phytoplankton (green waters), due to the consumption of CO_2_ by the algae (76). The phytoplankton is indirectly measured by the water transparence, and when maintained in equilibrium is an important additional food source for tilapia (77, 78). Besides that, algae may minimize the incidence of excessive light incidence in ponds (79). Thus, the maintenance of adequate levels of phytoplankton improves water quality and may provide greater comfort to fish in relation to environment luminosity, potentially improving tilapia welfare.

The external environment also influences fish welfare. For example, predation can be a cause of high mortality and stress in farmed fish. According to Broom (1), when exposed to predators, fish can show strong emergency adrenal responses and suppression of feeding behavior. Prevention measures for predator control include netting above or inside water, acoustic or visual devices (80, 81). On farm C, cages were screen-covered; however, birds were frequently present and often landed on that, suggesting that further actions are required. Anti-predator strategies are needed in excavated ponds, and because no actions were observed to minimize predation, this seems a relevant critical point for welfare assessment. An additional external deleterious effect on welfare was the excess of light exposure, since none of ponds had any shielding from direct sun light. A possible solution is the use of a fine mash above ponds or cages. The shading promoted by cage-covers can minimize the ultraviolet light and fright stress caused by overhead movement, both of which tend to reduce the risk of chronic stress and avoid predatory birds (74). Excessive lighting that occurs during massive capture operations is also a stressful factor, and when prolonged it becomes proportionally more deleterious to fish welfare.

Despite being a potential stress factor, air exposure, which occurred during pre-slaughter management, was trivialized at all facilities visited. The fact that tilapia can survive out of water for some period does not mean that it is a stress-free experience (47). The damages caused by air exposure depend on its duration and the fish species. European Food Safety Authority (EFSA) recommendation for trout is that air exposure be at most 10 s (82). This duration allowance for air exposure is critically inferior to the duration of at least 10 min observed in tilapia capture and pre-slaughter procedures on the studied farms. This confirms the urgent necessity of establishing welfare guidelines for tilapia farming.

In general, massive capture is associated with crowding, air and light exposure. The fish physiological response to these acute handling stressors is altered by previous long-term holding conditions (83). Crowding procedures are improved by efforts of reducing their duration and severity, in order to avoid additional suffering, stress, injuries, and mortality (5, 9, 84, 85). Considering the extremely high level and duration of crowding adopted during massive capture on farm A, that handling qualifies as unacceptable in terms of fish welfare, according to classification proposed by Noble et al. (26). Currently, there are management alternatives that avoid the contact of fish with air and light, through mechanical pumping (45). However, the main operational difficulty of mechanical pumping is to assure the removal of the whole population, including fish at the bottom of the tank and the injuries caused by suction pressure (47). Considering the traditional method, the color, size, and material of the sweep net may influence the stress caused to fish during capture, and the best choice of equipment depends on the situation (5). Lines and Spence (47) stated that welfare at capture can be improved by adopting and adapting procedures used for other species or developing completely new concepts or methods. Therefore, to mitigate stressors during tilapia capture, it is preferred to adopt the procedures and equipment that result in faster capture with less abrasive material, causing the lowest level of crowding, depending on circumstances.

Slaughter is considered one of the main critical points for fish welfare, mainly due to the lack of standardization and of legislation on fish humane slaughter practices (86, 87). The three main indicators of humane slaughter are the avoidance of excitement, pain, and suffering in the pre-slaughter handling, the loss of pain sensitivity within <1 s of the application of any aversive stunning or slaughter procedure, and its persistence until death (16). Methods of asphyxiation, decapitation with adherence of organs, and the pre-slaughter or slaughter with ice slurry observed in this work do not promote instant unconsciousness. Therefore, these methods cannot be considered humane (5, 9, 15). Including, there was resistance to allow the monitoring of these practices in at least six properties visited during the construction of this protocol. This may indicate that people are insecure about the adequacy of the practices adopted. According to Pedrazzani et al. (20), 87% of people interviewed in the town of Araucária, Southern Brazil, believed that fish are capable to feel pain and 85% that common slaughter methods cause suffering. Similar results were obtained by Rucinque et al. (88), who conducted an interview with highly educated citizens from Bogotá and Curitiba. From the participants, 79.7 and 71.8% perceived fish as sentient animals, and 76.0 and 72.0% believed that fish should be included in humane slaughter regulations, respectively. Webster (89) suggested that there is a gradual acceptance by farmers, scientists and veterinarians that farmed fish need to be treated in a humane and compassionate manner. As for slaughter, some efforts have been applied in the development of humane methods using electrical stunning for tilapia in Brazil; however, there is uncertainty regarding its effectiveness, due the lack of the monitoring of fish consciousness specific technical support and data registers during process.

Finally, general questions regarding overall welfare management are relevant. Farms in low-standard conditions are generally at greater risk of failing to respond to the basic welfare needs of farmed fish (90). This may be worse if there is a prevailing understanding that the animals are biologically able to cope with captive conditions, as is the common perception regarding tilapia. Even sturdier species may suffer with environmental challenges and it is the attribution of those responsible for the animals to always seek the best maintenance and management conditions. In this sense, tilapia welfare assessment may be used to identify critical welfare areas to be improved on farm (32). Some tilapia critical welfare points were common across all farms, even though the visited farms were diverse, including in terms of production systems adopted. These transversal critical welfare issues were the low rates of DO in water, the long duration of management for fish capture with exposure to air and crowding, and strongly aversive slaughter methods, which cannot be considered humane.

The lack of statistical significance between farms when comparing total scores is probably associated with the need for improvement in the integration of individual scores into a final overall welfare category to each farm. This is a recurrent difficulty in animal welfare assessment, to which even more refined integration methods, such as those proposed by the Welfare Quality protocols (22, 23, 91), have not yet provided a completely satisfactory solution (92). Even though the main goal of our work was to determine a robust protocol containing major critical welfare points in different scenarios, we believe further research into the integration of individual scores to produce an overall welfare assessment warrants further studies.

## Applications and Conclusions

The identification of critical tilapia welfare issues seems essential for farmers to adopt preventive management actions (93). For example, some conditions such as gill and fin problems are affected not just by handling, but also by the confinement conditions (5, 44) and seem to deserve higher levels of attention. Then, the regular use of a tilapia welfare assessment protocol becomes an important management tool. Additionally, the protocol is open to the inclusion of new welfare indicators, and the enrichment of the list of behavioral indicators is urgent, especially indicators that allow for a closer observation of tilapia behavior throughout their complete life cycles. This priority is evident, as the understanding of welfare depends inherently on the direct observation of the individuals and animal behavior is a major form for the expression of emotions and feelings in non-verbal species (94). Despite the challenge of high turbidity of pond water, methods for underwater behavioral assessment must be developed in order to obtain a better understanding of specific issues such as the hierarchical relationship between fish, especially during the feeding; the occupation of the water column in terms of cage or pond area that is actually useful for the tilapias and its implications for natural swimming behavior; the proper calculation of density; and the development of environmental enrichment techniques (90). In few words, in order to be effective in the monitoring and enhancement of animal welfare, animals must be seen throughout their lives.

It seems relevant to emphasize that the practical application of this first protocol, even though it is not exhaustive, will allow the producer to be closer to the animals, just as it happens with terrestrial vertebrates. This strengthening of communication may be an ally to the prevention of diseases and control of other potential problems related to water quality, external environment, and inadequate management, thus minimizing the harmful effects caused by the productive systems to the welfare of tilapia. This may be an initial step for a tilapia welfare strategy, where the prioritization of critical points, implementation of corrective actions and monitoring of the results is part of a permanent welfare management program. A final important remark is the fact that the protocol also lends itself to adaptation into a mobile application, which may further facilitate on-farm use and promote its adoption.

Our results suggest that a tilapia welfare assessment routine may be in place with a single protocol, which seems effective in different farming realities and feasible for farm staff use. Furthermore, the developed protocol has shown relative discriminating power, high on-field feasibility and a clear role in determining critical points in tilapia welfare, which in turn may guide management decisions. Considering the challenges presented for further improvements to the protocol, we believe that the format presented, which is compatible with and close to that of other species welfare assessment protocols with longer history of use and refinements may help the identification of best future approaches. Finally, refinements to the protocol are welcome in relation to the integration of the indicators into a single final score for each property, in addition to the continuous refinement of the existent indicators and the inclusion of new tilapia welfare indicators as they become available.

## Data Availability Statement

The raw data supporting the conclusions of this article will be made available by the authors, without undue reservation.

## Ethics Statement

The animal study was reviewed and approved by Animal Use Ethics Committee of the Agricultural Campus, Federal University of Paraná (No. 083/2019).

## Author Contributions

MQ: funding acquisition. AP, CM, and MQ: study conception and design. AP, FB, and ES: performance of the data collection and tabulation. AP: statistical modeling and data analyses. AP and CM: preparation of the manuscript. MQ and CM: project coordination. All authors reviewed and approved the final version of manuscript.

## Conflict of Interest

MQ and FB were employed by FAI Farms. The remaining authors declare that the research was conducted in the absence of any commercial or financial relationships that could be construed as a potential conflict of interest.
